# Quality of Data Recording and Antimicrobial Use in a Municipal Veterinary Clinic in Ghana

**DOI:** 10.3390/tropicalmed8110485

**Published:** 2023-10-26

**Authors:** Cletus Kubasari, Wisdom Adeapena, Robinah Najjemba, George Kwesi Hedidor, Raymond Lovelace Adjei, Grace Manu, Collins Timire, Samuel Afari-Asiedu, Kwaku Poku Asante

**Affiliations:** 1 Kintampo Health Research Centre, Research and Development Division, Ghana Health Service, Kintampo P.O. Box 200, Ghana; wisdomadeapena@kintampo-hrc.org (W.A.); grace.manu@kintampo-hrc.org (G.M.); samuel.afari-asiedu@kintampo-hrc.org (S.A.-A.); kwakupoku.asante@kintampo-hrc.org (K.P.A.); 2 Public Health Consultant, 1202 Geneva, Switzerland; robinahnajjemba@yahoo.co.uk; 3 Ghana Country Office, World Health Organization, Accra P.O. Box MB 142, Ghana; hedidorg@who.int; 4 Council for Scientific and Industrial Research-Animal Research Institute, Accra P.O Box 20, Ghana; raymondadjei@csir.org.gh; 5 International Union Against Tuberculosis and Lung Diseases, 75006 Paris, France; collinstimire2005@yahoo.com

**Keywords:** antimicrobial resistance, veterinary service, data quality, antimicrobial use, prescription practice, Ghana, SORT IT, operational research

## Abstract

The recording of antimicrobial use data is critical for the development of interventions for the containment of antimicrobial resistance. This cross-sectional study assessed whether dissemination activities and recommendations made after an operational research (OR) study in 2021 resulted in better data recording and improved the use of antimicrobials in a rural veterinary clinic. Routinely collected data from treatment record books were compared between 2013 and 2019 (pre-OR) and from July 2021 to April 2023 (post-OR). The most common animals presenting for care in the the pre – and post OR periods were dogs (369 and 206, respectively). Overall, antimicrobial use in animals increased from 53% to 77% between the two periods. Tetracycline was the most commonly used antimicrobial (99%) during the pre-OR period, while Penicillin-Streptomycin was the most commonly used antimicrobial (65%) during the post-OR period. All animals that received care at the clinic were documented in the register during both periods. Whereas the diagnosis was documented in 269 (90%) animals in the post-OR period compared to 242 (47%) in the pre-OR period, the routes and dosages were not adequately recorded during the both periods. Therefore, the quality of data recording was still deficient despite the dissemination and the recommendations made to some key stakeholders. Recommendations are made for a standardized antimicrobial reporting tool, refresher training, and continuous supervisory visits to the clinic.

## 1. Introduction

The World Health Organization, in 2019, ranked antimicrobial resistance (AMR) among the top ten (10) global public health emergencies that require urgent and strategic measures in order to mitigate the increasing threat [1]. One of the recommended effective strategies for tackling AMR globally is the adoption of the “One Health” approach, which encourages the harmonization of efforts among different institutions and experts across different levels of society. The aim of the harmonized effort of the “One Health” approach is to address AMR in humans, animals, and the environment, considering that the health and well-being of all three are interconnected [2].

The quality of prescription data recorded by veterinary healthcare providers is critical in the development of the national standards and policies required to contain the development of antimicrobial resistance (AMR) [3]. These data contribute to the understanding of the factors that contributes to antimicrobial stewardship, which is essential for monitoring and tracking the use of antimicrobials in veterinary facilities [4]. Accurate, complete, and standardized data recording formats are essential for effectively monitoring the consumption of antimicrobials in veterinary health facilities [5]. Poor data quality can also affect the analysis and interpretation of data, resulting in undesirable outcomes [4,6].

Despite persistent efforts to establish antimicrobial use surveillance programs in many Low- and Middle-Income Countries (LMICs), accessible data are still inadequate. The data used for antimicrobial stewardship programs implemented in Africa for continuous quality improvement is largely obtained from global AMR control initiatives [7]. These may vary from one country to another as a result of the different methodologies and tools used [3,7].

Many LMICs, including Ghana, either lack or have inadequate uptake of context-specific policy frameworks [8,9]. These frameworks may include National Action Plans (NAPs) that clearly outline objectives and strategies for addressing the issues related to antimicrobial resistance specific to a country’s veterinary medicine sector, antimicrobial prescription guidelines on how antimicrobials should be prescribed for the management of diseases in animals, and education and training for veterinary prescribers on the best practices for prescribing and dispensing antimicrobials [3,8]. In 2017, 44 out the 54 African countries submitted reports on antimicrobial use to the World Organization for Animal Health (WOAH, previously OiE). The estimates of data coverage were however seen to be lowest among African countries and the data was also seen to have issues related to quality [10].

In addition, many LMICs have a large range of untrained antimicrobial prescribers, and there is easy access to antimicrobials that can be purchased over the counter and without prescription [11,12]. This is coupled with large numbers of counterfeit medicines, especially in the private sector, due to weak implementation of regulations [8,9].

The burden of AMR in animals is difficult to measure, especially in LMICs, due to the unreliable and poor reporting of data for stewardship programs, AMR surveillance programs, and monitoring systems [13,14,15]. The optimized use of antimicrobials can be assessed by monitoring adherence to specific treatment guidelines and documenting prescription practices related to the appropriate use of antimicrobials and the correct dosage, frequency of administration, and duration of treatment [16]. In this regard, the World Organization for Animal Health (WOAH, formerly known as OIE) provides guidelines for the use of antimicrobials and the monitoring of antimicrobial resistance in animal health [17]. The guidelines provide a well-established data collection system that, if followed, produces representative and validated data that are critical for tracking and assessing treatment outcomes in animal health [18].

In Ghana, an operational research study conducted by Adeapena et al. in 2021 observed poor data documentation practices and high antimicrobial use at a rural veterinary clinic. Diagnoses were missing in 53% of records, dosages were missing in 38% of records, and the route of administration of antimicrobials was not recorded in 69% of records. Tetracycline accounted for 99% of all the antimicrobials used [19]. This study, conducted through the Structured Operational Research Training Initiative (SORT IT), led to various recommendations to improve the situation [19].

The published paper, elevator pitches, plain-language handouts, and lightening and technical PowerPoint presentations were used to disseminate the findings and make recommendations to key stakeholders for action to improve data recording and prescription practices.

The current study was conducted as a follow-up to assess whether the dissemination activities and recommendations made to relevant stakeholders in the veterinary services, including the Veterinary Service Directorate, Pharmacy Council, Food and Drugs Authority, Researchers, and the Ministry of Health, contributed to improvement in the quality of data recording and the use of antimicrobials at the veterinary clinic between July 2021 and April 2023 compared to the previous period. Specifically, we compared the proportion of animals treated with antimicrobials and assessed changes in the quality of data recording in terms of the completeness of data on diagnosis, the type of antimicrobial used, the antimicrobial dosage, and the route of administration for all animals that received antimicrobials.

## 2. Materials and Methods

### 2.1. Study Design

This was a cross-sectional study using routinely collected data from treatment record books between 2013 and 2019 (pre-SORT IT) and from July 2021 to April 2023 (post-SORT IT).

### 2.2. Study Setting

#### 2.2.1. General Setting

Ghana is bordered on the east, west, and north by the republics of Togo, Côte d’Ivoire, and Burkina Faso, respectively. The livestock sector contributed 5.4 percent of Ghana’s agricultural Gross Domestic Product (GDP) growth in 2019 [20]. Veterinary services are under the regulation of the Veterinary Service Directorate (VSD), which is under the Ministry of Food and Agriculture. There are approximately 256 veterinary clinics nationwide [19]. The Veterinary Training Institute (VTI) under the Veterinary Services Directorate of Ghana is responsible for training veterinary professionals across the 4 veterinary training schools in the country. There are 11 veterinary offices in the Bono East region (https://vetservicesgh.org/index.php/about-us/offices, accessed on 4 August 2023)

Through the Regional Directorate, a national information management system compiles monthly and quarterly reports from district officials. Every district has a District Veterinary Officer and several veterinary paraprofessionals. There is currently no national electronic database; hence, all reports are submitted as hard paper copies [19]. The VSD, under the Ministry of Food and Agriculture, monitors the supply of veterinary pharmaceuticals using a coordinated system with the assistance of private veterinary pharmaceutical firms [21]. The VSD is in charge of making veterinary medications and vaccines available through both public and private procurement methods. The VSD follows the Economic Community of West African States (ECOWAS) veterinary pharmacy protocol [19]. These pharmaceuticals are provided to several Regional Veterinary Directorates that then distribute them to districts and other private veterinary medical shops throughout the country [19].

#### 2.2.2. Specific Setting

The Kintampo North Municipality is situated in the middle belt of Ghana. The inhabitants’ livelihood is mainly farming, including animal husbandry for both commercial and domestic purposes. The population of Kintampo Municipality is 228,634, representing 35.2 percent of the region’s total population [22].

The Kintampo Municipal Veterinary Clinic was chosen for this study because this is where the previous study was conducted, and it is the only clinic providing veterinary services to over 100 urban and rural communities within the municipality. The veterinary technologist is supported by other para-veterinary officers to provide routine clinical and surgical services within the clinic space and outreach activities to larger animal farms. The clinic lacks animal laboratory services, so disease diagnosis is still based on the clinical experience of the staff. Some of the injectable antimicrobials stocked at the clinic include Penicillin–Streptomycin, Oxytetracycline, Enrofloxacin, Metronidazole, and Gentamicin. In the case of stock-outs, the farmers are referred to the only private veterinary medicine retailer in the municipality, which provides alternative access to veterinary medicines. Besides clinical and surgical services, the clinic’s staff conduct annual community education and vaccination campaigns to educate the community about zoonotic infections and domestic and farm animal care.

**Figure 1:**
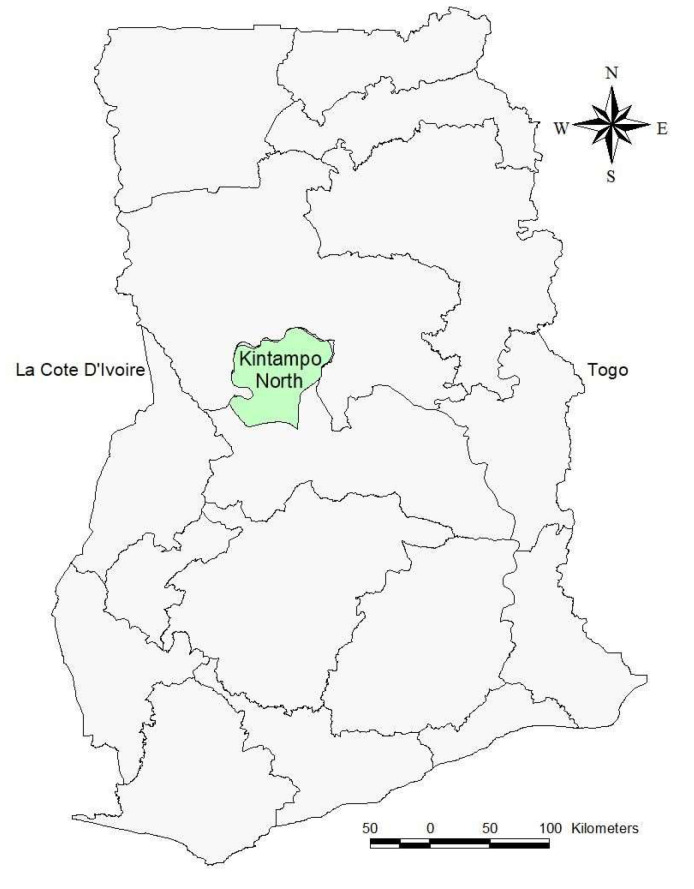
Map of Kintampo [19].

Treatments are manually recorded in a treatment register. Good documentation practices should include the type of animal (breed), diagnosis, type of treatment (medication), route of administration, and dosage [23]. Monthly reports are generated and sent to the Regional Directorate as part of the monitoring and evaluation system as hard copies [19]. The Figure 1 below is a map of Ghana showing the study area.

### 2.3. Study Population

The study population consisted of animals that received veterinary care from the Kintampo Veterinary Clinic from 2013 to 2019 (pre-operational-research (pre-OR) period)) and from July 2021 to April 2023 (post-operational-research (post-OR) period).

### 2.4. Data Sources

Objective 1, was to describe the dissemination activities, the recommendations made, and the actions taken to improve the completeness of the recording of prescription data post-OR. Data for Objective 1, were collected from the Principal Investigator (PI) of the pre-OR study. Objectives 2 and 3, which aimed to compare the proportion of animals treated with antimicrobials at the Kintampo Municipal Veterinary Clinic and assess changes in the quality of data recording in terms of the completeness of data, diagnosis, antimicrobial type, dosage, and route of administration respectively. The data for objective 2 and 3 were retrieved from the Kintampo Municipal Veterinary Clinic treatment register and compared with data from the pre-OR period [19]. 

### 2.5. Data Collection and Validation

Data for the post-OR period were double-entered into MS Excel and validated by cross-checking records in the treatment register.

### 2.6. Data Analysis and Statistics

For the pre-OR period, data from the SORT IT monitoring and evaluation phase were compiled and complemented with information from the PI of the pre-OR study. These were presented as a single-line list of activities undertaken to disseminate the results and the actions taken on the recommendations.

For the post-OR period, data were imported from MS Excel into EpiData analysis v 3.1 software (EpiData Association, Odense, Denmark) for analysis. Categorical variables were summarized using frequencies and proportions, and results were presented in tables. Differences in the proportions of animals receiving antimicrobials between the two study periods (2013–2019 and 2021–2023) were assessed using the chi-square test. The level of significance was set at *p* < 0.05 along with 95% confidence intervals (95% CI).

## 3. Results

### 3.1. Documentation of Dissemination and Recommendations Made Post-OR

A SORT IT module was conducted in November 2021 to develop the knowledge management and communication skills of researchers. The outputs of this module were the development of the following tools: (1) a communication plan to target key stakeholders, policy, and decision makers; (2) a PowerPoint presentation to be used at conferences; (3) a one-page plain-language handout of the key findings and messages; (4) a one-minute oral presentation (an elevator pitch); and (5) a poster presentation of the research findings. These tools were used for the communication and dissemination of the research findings to stakeholders, noting the mode of delivery, audience, place, and time. The actions taken post-dissemination and the recommendations made using these tools were documented and verified with the PI of the first publication. Refer to Table 1 and Table 2 for the presentation of these results.

### 3.2. Types and Proportions of Animals That Were Registered for Veterinary Care during Pre-OR and Post-OR Periods

During the pre-OR period (2013–2019), a total of 513 animals presented at the Kintampo Veterinary Clinic, compared to 299 animals during the post-OR period (July 2021–April 2023). Dogs (n = 369 (72%) and n = 206 (69%)) constituted the most common animals during both time periods, followed by goats (n = 67 (13%) and n = 64 (21%)). Refer to Table 3 for the presentation of the results.

### 3.3. Documentation of Diagnosis, Antimicrobials Prescribed, Dosage, and Route of Administration of Antimicrobials at the Kintampo Municipal Veterinary Clinic (From July 2021 to April 2023)

In total, 513 and 299 animals received care at the Kintampo Veterinary Clinic during the pre-OR (2013–2019) and post-OR (2021–2023) periods, respectively, and the animal type was recorded in every case during the two study periods. Of these, 269 (90%) had their diagnosis documented during the post-OR period, compared to 242 (47%) during the pre-OR period. Also, 231 (77%) of the antimicrobials given were recorded in the post-OR period, compared to 273 (53%) in the pre-OR period. Refer to Table 4 for the presentation of the results.

### 3.4. Antimicrobials Prescribed at the Clinic during the Pre-OR and Post-OR Study Periods

During the pre-OR (2013–2019) period, Oxytetracycline was the most commonly prescribed antimicrobial (n = 272 (99%) versus n = 74 (32%)), whereas during the post-OR period, Penicillin-Streptomycin (Pen-Strep) was the most commonly prescribed antimicrobial (n = 0 (0%) versus n = 150 (65%)). Refer to Table 5 below for the presentation of the results.

Overall, 273/513 (53%) animals received antimicrobials during the pre-OR period, compared to 231/299 (77%) animals that received antimicrobials during the post-OR period. In addition, 191 (52%) versus 159 (77%) dogs received antimicrobials during the two time periods, respectively. Refer to Table 6 for the presentation of the results.

## 4. Discussion

This operational research study showed that a large proportion of animals were treated with antimicrobials before (pre-OR) and after (post-OR) implementing measures that sought to promote antibiotic stewardship in one veterinary clinic at the Kintampo North Municipality clinic. However, the highest proportion of antimicrobials were prescribed during the post-OR period as compared to the pre-OR period. Penicillin-Streptomycin was the most commonly used antimicrobial during the post-OR period, compared to Tetracycline during the pre-OR period [19]. There were some gaps in the quality of the prescription data with regards to the dosage and route of administration of antimicrobials at both study points [19].

The findings of the frequent use of antimicrobials at the veterinary clinic are comparable to those reported in the pre-OR [19] period and antimicrobial use in a study conducted among human populations in the same study area [24]. They are also comparable to the findings of studies conducted in Rwanda and Tanzania [25,26]. The high rates of prescription and use of antimicrobials by veterinary healthcare providers may be a result of poor antimicrobial surveillance systems and weak or non-existent regulations for monitoring the use of antimicrobials in animal healthcare systems in Ghana and other LMICs [11,27] as compared to developed countries in Europe and Asia [19]. This may be further compounded by a wide range of untrained antimicrobial prescribers and easy access to antimicrobials, which can be purchased over the counter in these LMICs [8,9]. Immediate actions need to be taken in order to increase the awareness of antimicrobial resistance and to promote behavior change through various media that target different professionals in animal and human health and in agricultural practice as well as among other users of antimicrobials [28]. Discussing the use of antimicrobials and antimicrobial resistance as parts of academic activities and during professional training and capacity-building programs of health providers would help prescribers to better understand and appreciate the magnitude of the situation and how they can best contribute to mitigating the growing threat [28,29,30].

The enforcement of existing regulations and guidelines on antimicrobial use by the appropriate institutions and agencies in Ghana would also help in controlling the excessive use of antimicrobials across different sectors [31].

The most commonly prescribed antimicrobial during the post-OR period was Pen-Strep, a combination of penicillin and streptomycin. This finding, however, contrasts with the results of the pre-OR study conducted in 2021 and another study in Nigeria where tetracycline was the most commonly prescribed antimicrobial [19,32]. These differences in the two study periods may have resulted from factors such as the availability (stock levels) of the various antimicrobials observed or possible stock-outs of other antimicrobials. The availability and prescription of antimicrobials in veterinary facilities in many resource-poor settings are influenced by many factors that include cost and affordability, budget allocations for the sector, and supply chain management systems [33].

Also, as shown by other studies conducted to assess some of the non-clinical factors that influence the prescription of antimicrobials by veterinarians, individual prescriber preferences could be a factor for the differences since there was a change in the overall management of the veterinary clinic during post-OR period [34,35].

With regards to the quality of prescription data, there were more gaps in the dosage and route of administration of the various antimicrobials in the post-OR period as compared to the pre-OR period. Despite the fact that the dosages of antimicrobials depend on the animals’ ages and weights [36], these were not recorded in the treatment record books of the clinic for almost all cases during the post-OR period. There were also gaps in the data on the route of administration. This observation could be because most of the antimicrobials stocked at the clinic are for administration by the parenteral route, in which case the prescriber may have taken it as obvious that the person administering the antimicrobial would know the appropriate route to use. This, however, not standard practice, as it puts the life of the animal in danger when an inappropriate route of administration is used [37].

The findings mentioned above are comparable to the results of the baseline study conducted in 2021, where the data for these variables were also reported as incomplete [19]. This low quality of data recording may be attributed to a lack of appropriate tools such as standard treatment registers, including electronic data systems, which are the most reliable [36]; high staff turnover; and probably a lack of frequent supportive supervision by a national regulatory body such as the Food and Drugs Authority [27]. A key requirement for effectively monitoring the use of antimicrobials in stewardship programs is accurate and complete data [38]. In a traditional paper-based data recording system, the issue of the inadequate recording of important treatment information (missing data) has been previously reported where the dosage, the route of administration, and the duration of treatment, coupled with illegible prescriber handwriting, were found to be common [39]. Therefore, to ensure the comprehensive documentation of data, harmonized electronic data recording is ideal for improving the accuracy, completeness, and overall data integrity since healthcare providers can easily input data directly and reduce errors. These data can also be accessed in real time to aid in decision making [38].

There are some important limitations to our study. First, a key limitation of this study is that we only assessed the documentation practices of a single veterinary facility, so the results may not be reflective of practices in other veterinary facilities across the country.

Secondly, we cannot directly associate the changes in this current study to the dissemination activities that were carried out after the first OR study. This is because the research team could not share the pre-OR study’s findings with the entire clinic staff and only shared them with the veterinary officer who was overseeing the activities of the unit at the time. Also, the periods of data collection for both studies varied: while the pre-OR study analyzed data for a period of 5 years, the post-OR study analyzed 3 years of data. Additionally, data on the medical conditions among the animals receiving antimicrobials were inadequate, as this information was either missing or lacked sufficient detail, and thus we could not measure the extent of the inappropriate antimicrobial use in the clinic. 

## 5. Conclusions

The study’s findings indicate that antimicrobial use at the clinic during both reporting periods (pre-OR and post-OR) was high, although a greater proportion of antimicrobials were prescribed during the post-OR period as compared to the pre-OR period. There was also a significant improvement in the documentation of the reasons for which antimicrobials were prescribed (diagnosis) for treatment in the post-OR period. However, the documentation of the routes of administration, the dosage of antimicrobials prescribed for treatment, and the duration of treatment was still poor during the post-OR study period, and this needs to be improved.

## Figures and Tables

**Table 1:** Dissemination details following the operational research study conducted in 2021.

Mode of Delivery	To Whom	Where	When
PowerPoint presentation Plain-language handout Published article Published article uploaded to the institution’s website	Kintampo Health Research Centre Scientific Technical Committee.	KHRC conference room	February and August 2022
PowerPoint presentation Plain-language handout	Ghana AMR platform	Virtual (Zoom)	May 2021
		Conference hall of AH hotel	May 2021
Open-access publication	Global [19] (4587 views and cited by 4).	MDPI (Tropical Medicine and Infectious Disease)	July 2021
Twitter, LinkedIn, Facebook, and WhatsApp	Global	Social media	July 2021
PowerPoint presentation Plain-language handout Published article	Key stakeholders (Veterinary Service Directorate, Pharmacy Council, Food and Drugs Authority, researchers, and policy makers)	Conference room of Mensvic Hotel, Accra.	September 2021

**Table 2:** List of recommendations from the pre-operational research study for improving data recording and antimicrobial use and the status of implementation [19].

Recommendation	Action Status	Details of Action (When and What)
Sensitization of veterinary staff about the risks of misuse of antimicrobials and the lack of quality data on antimicrobial use for monitoring purposes	Ongoing	In May 2021, key stakeholders including the head and staff of the veterinary clinic, the Veterinary Service Directorate, Food and Drugs Authority, and staff of the Pharmacy Council of Ghana were sensitized about antimicrobial resistance and good documentation/recording practices.
Training of veterinary staff at the clinic	On hold	The staff of the veterinary clinic will be trained in appropriate record keeping. Arrangements have been made to engage the staff at the clinic again.
Further research on the use of antimicrobials in animals across all veterinary clinics in the country to give a holistic reflection of antimicrobial use and serve as country-wide baseline data	Partial implementation by the Ghana Veterinary Service Directorate	Developed a proposal on the need for a national survey.
Develop a Ghana-specific standard treatment guideline for animal health practitioners	Decision taken by the Ghana Veterinary Service Directorate	Standard treatment guidelines for antimicrobial use in animals were developed by the Veterinary Service Directorate, is yet to launch this.
Electronic system of data capture	Partial implementation by the Ghana Veterinary Service Directorate	Software was piloted using tablets to capture data in some selected veterinary clinics in Accra, but this has yet to be carried out in rural parts of Ghana.

**Table 3:** Types, numbers, and proportions of animals that received care at the Kintampo Municipal Veterinary Clinic during the pre-OR (2013–2019) and post-OR (July 2021–2023) periods.

	2013–2019		2021–2023	
Animal Type	Number	% *	Number	% *
Dogs	369	71.9	206	68.9
Goats	67	13.1	64	21.4
Sheep	57	11.1	20	6.7
^ Other animals	20	3.9	9	3.0
Total	513	100.0	299	100.0

Sources of data: Adeapena et.al., 2021 [19], and from Fieldwork, 2023 respectively. ^ Other = combination of cattle, monkeys, poultry, rabbits, and cats, which were very few during both the pre-OR (2013–2019) and post-OR (July 2021–April 2023) study periods. * Column percentages: proportions of animals that received care at the Kintampo Municipal Veterinary Clinic during the pre-OR (2013–2019) and post-OR (July 2021–2023) periods.

**Table 4:** Documentation of treatment records during pre-OR and post-OR periods.

	Pre-OR (2013–2019)			Post OR (2021–2023)			
Record Documentation	Total	n	(%) *	Total	n	(%) *	*p*-Value
Diagnosis specified	513	242	(47.2)	299	269	(90.0)	<0.0001
Antimicrobial prescribed	513	273	(53.2)	299	231	(77.3)	<0.0001
Route of administration documented	273	85	(31.1)	231 ^	1	(0.2)	<0.0001
Dosage documented	273	170	(62.3)	231 ^	1	(0.2)	<0.0001

Sources of data: Adeapena et.al., 2021 [19], and from Fieldwork, 2023 respectively. ^ = One animal received two different antimicrobials during the post-SORT IT period (July 2021–2023). * Row Percentages: completeness of treatment records from July 2021 to April 2023 at the Kintampo Veterinary Clinic.

**Table 5:** The proportions of antimicrobials that were prescribed at the Kintampo Municipal Clinic during the pre-OR (2013–2019) and post-OR periods.

	2013–2019		2021–2023	
Antimicrobial Prescribed	Number	%*	Number	%*
Oxytetracycline	272	99.6	74	32.0
^ Pen-Strep	0	0.0	150	64.9
Enrofloxacin	0	0.0	5	2.2
Penicillin	1	0.4	0	0.0
Gentamicin	0	0.0	1	0.4
Metronidazole	0	0.0	1	0.4
Total	273	100.0	231	100.0

Sources of data: Adeapena et.al., 2021 [19], and from Fieldwork, 2023 respectively. * Column percentages: the proportions of antimicrobials that were prescribed at the Kintampo Municipal Clinic during the pre-OR (2013–2019) and post-OR periods. ^ Pen-Strep = a combination of penicillin and streptomycin.

**Table 6:** The animal types, numbers, and proportions that received antimicrobials at the Kintampo Veterinary Clinic during the pre-OR and post-OR periods.

2013–2019				2021–2023			
Animal Type	Total	Received Antimicrobial	% *	Total	Received Antimicrobial	% *	*p*-Value
Dogs	369	191	52	206	159	77	<0.0001
Goats	67	36	54	64	50	78	0.003
Sheep	57	36	63	20	16	80	0.14
^ Other	20	10	50	10	5	50	0.5

Sources of data: Adeapena et.al., 2021 [19], and from Fieldwork, 2023 respectively. * Row percentages: the proportions of animals that received antimicrobials during the two time periods. ^ Other = combination of cattle, monkeys, poultry, rabbits, and cats, which were very few during the pre-OR (2013–2019) and post-OR (July 2021–2023) periods.

## Data Availability

The data are available on request from the corresponding author.

## References

[1] Sun R., Yao T., Zhou X., Harbarth S., Lin L. (2022). Non-biomedical factors affecting antibiotic use in the community: A mixed-methods systematic review and meta-analysis. Clin. Microbiol. Infect..

[2] Adisasmito W.B., Almuhairi S., Behravesh C.B., Bilivogui P., Bukachi S.A., Casas N., Becerra N.C., Charron D.F., Chaudhary A., Zanella J.R.C. (2022). One Health: A new definition for a sustainable and healthy future. PLoS Pathog..

[3] Kiggundu R., Lusaya E., Seni J., Waswa J., Kakooza F., Tjipura D., Kikule K., Muiva C., Joshi M.P., Stergachis A. (2023). Identifying and addressing challenges to antimicrobial use surveillance in the human health sector in low-and middle-income countries: Experiences and lessons learned from Tanzania and Uganda. Antimicrob. Resist. Infect. Control.

[4] Hammer A., Wagner A., Rieger M.A., Manser T. (2019). Assessing the quality of medication documentation: Development and feasibility of the MediDocQ instrument for retrospective chart review in the hospital setting. BMJ Open.

[5] WHO (2018). Global Antimicrobial Resistance Surveillance System (GLASS) Report: Early Implementation 2016–2017. https://www.who.int/publications/i/item/9789241513449.

[6] Tubaishat A., Tawalbeh L.I., AlAzzam M., AlBashtawy M., Batiha A.-M. (2015). Electronic versus paper records: Documentation of pressure ulcer data. Br. J. Nurs..

[7] Waswa J., Kiggundu R., Konduri N., Kasujja H., Lawry L.L., Joshi M.P. (2023). What is the appropriate antimicrobial use surveillance tool at the health facility level for Uganda and other low-and middle-income countries?. J. Glob. Antimicrob. Resist..

[8] Cox J.A., Vlieghe E., Mendelson M., Wertheim H., Ndegwa L., Villegas M.V., Gould I., Hara G.L. (2017). Antibiotic stewardship in low-and middle-income countries: The same but different?. Clin. Microbiol. Infect..

[9] Kakkar A.K., Shafiq N., Singh G., Ray P., Gautam V., Agarwal R., Muralidharan J., Arora P. (2020). Antimicrobial stewardship programs in resource constrained environments: Understanding and addressing the need of the systems. Front. Public Health.

[10] Mikecz O., Pica-Ciamarra U., Felis A., Nizeyimana G., Okello P., Brunelli C. (2020). Data on antimicrobial use in livestock: Lessons from Uganda. One Health..

[11] Afari-Asiedu S., Kinsman J., Boamah-Kaali E., Abdulai M.A., Gyapong M., Sankoh O., Hulscher M., Asante K.P., Wertheim H. (2018). To sell or not to sell; the differences between regulatory and community demands regarding access to antibiotics in rural Ghana. J. Pharm. Policy Pract..

[12] Sulis G., Gandra S. (2021). Access to antibiotics: Not a problem in some LMICs. Lancet Glob. Health.

[13] Ducrot C., Hobeika A., Lienhardt C., Wieland B., Dehays C., Delabouglise A., Bordier M., Goutard F., Patel E., Figuié M. (2021). Antimicrobial resistance in Africa—How to relieve the burden on family farmers. Emerg. Infect. Dis..

[14] Hardefeldt L.Y., Gilkerson J., Billman-Jacobe H., Stevenson M., Thursky K., Bailey K., Browning G. (2018). Barriers to and enablers of implementing antimicrobial stewardship programs in veterinary practices. J. Vet. Intern. Med..

[15] Hosain M.Z., Kabir S.L., Kamal M.M. (2021). Antimicrobial uses for livestock production in developing countries. Vet. World.

[16] Mendelson M., Morris A., Thursky K., Pulcini C. (2020). How to start an antimicrobial stewardship programme in a hospital. Clin. Microbiol. Infect..

[17] Pinto Ferreira J., Gochez D., Jeannin M., Magongo M.W., Loi C., Bucher K., Moulin G., Erlacher-Vindel E. (2022). From OIE standards to responsible and prudent use of antimicrobials: Supporting stewardship for the use of antimicrobial agents in animals. JAC-Antimicrob. Resist..

[18] WHO (2018). WHO Report on Surveillance of Antibiotic Consumption: 2016–2018 Early Implementation. https://apps.who.int/iris/bitstream/handle/10665/277359/9789241514880-eng.pdf.

[19] Adeapena W., Afari-Asiedu S., Najjemba R., Griensven J.v., Delamou A., Ohene Buabeng K., Poku Asante K. (2021). Antibiotic use in a municipal veterinary clinic in Ghana. Trop. Med. Infect. Dis..

[20] Rebased G.S.S. Rebased 2013–2019 annual gross domestic product. Ghana Statistical Service. 2020, 11 April 2020 Edition. https://statsghana.gov.gh/gssmain/storage/img/marqueeupdater/Annual_2013_2019_GDP.pdf.

[21] Luseba D., Rwambo P. Review of the policy, regulatory and administrative framework for delivery of livestock health products and services in Eastern and Southern Africa. February 2015 edition. https://www.researchgate.net/profile/Dibungi-Luseba/publication/339237659_Review_of_the_policy_regulatory_and_administrative_framework_for_delivery_of_livestock_health_products_and_services_in_West_and_Central_Africa_Prepared_for_GALVmed_by/links/5e455ab592851c7f7f367bea/Review-of-the-policy-regulatory-and-administrative-framework-for-delivery-of-livestock-health-products-and-services-in-West-and-Central-Africa-Prepared-for-GALVmed-by.pdf.

[22] Ghana Statistical Service (2021). Ghana 2021 Population and Housing Census General Report.

[23] Fajt V.R., Lehenbauer T.W., Plummer P.J., Robbins R.C., Scheftel J.M., Singer R.S., Canon A.J., Frey E., Gaunt P.S., Papich M.G. (2022). A call to action for veterinarians and partners in animal health to collect antimicrobial use data for the purposes of supporting medical decision-making and antimicrobial stewardship. J. Am. Vet. Med. Assoc..

[24] Afari-Asiedu S., Oppong F.B., Tostmann A., Ali Abdulai M., Boamah-Kaali E., Gyaase S., Agyei O., Kinsman J., Hulscher M., Wertheim H.F. (2020). Determinants of inappropriate antibiotics use in rural central Ghana using a mixed methods approach. Front. Public Health.

[25] Kimera Z.I., Frumence G., Mboera L.E., Rweyemamu M., Mshana S.E., Matee M.I. (2020). Assessment of drivers of antimicrobial use and resistance in poultry and domestic pig farming in the Msimbazi river basin in Tanzania. Antibiotics.

[26] Manishimwe R., Nishimwe K., Ojok L. (2017). Assessment of antibiotic use in farm animals in Rwanda. Trop. Anim. Health Prod..

[27] Yevutsey S.K., Buabeng K.O., Aikins M., Anto B.P., Biritwum R.B., Frimodt-Møller N., Gyansa-Lutterodt M. (2017). Situational analysis of antibiotic use and resistance in Ghana: Policy and regulation. BMC Public Health.

[28] WHO (2015). Global Action Plan on Antimicrobial Resistance. https://apps.who.int/iris/bitstream/handle/10665/193736/9789241509763_eng.pdf.

[29] Mutua F., Sharma G., Grace D., Bandyopadhyay S., Shome B., Lindahl J. (2020). A review of animal health and drug use practices in India, and their possible link to antimicrobial resistance. Antimicrob. Resist. Infect. Control.

[30] Odoi A., Samuels R., Carter C.N., Smith J. (2021). Antibiotic prescription practices and opinions regarding antimicrobial resistance among veterinarians in Kentucky, USA. PLoS ONE.

[31] Boamah V.E., Agyare C., Odoi H., Dalsgaard A. (2016). Practices and Factors Influencing the Use of Antibiotics in Selected Poultry Farms in Ghana. https://www.researchgate.net/profile/Christian-Agyare/publication/304822555_Practices_and_factors_influencing_the_use_of_antibiotics_in_selected_poultry_farms_in_Ghana/links/577bd0cd08ae355e74f16464/Practices-and-factors-influencing-the-use-of-antibiotics-in-selected-poultry-farms-in-Ghana.pdf.

[32] Aworh M.K., Kwaga J.K.P., Okolocha E.C. (2021). Assessing knowledge, attitude, and practices of veterinarians towards antimicrobial use and stewardship as drivers of inappropriate use in Abuja, Nigeria. One Health Outlook.

[33] Isabel Frost J.C., Joshi J., Faure K., Laxminarayan R. (2019). Access Barriers to Antibiotics. https://onehealthtrust.org/wp-content/uploads/2019/04/AccessBarrierstoAntibiotics_CDDEP_FINAL.pdf.

[34] Pramil T., Rajiv A., Gaurav G. (2014). Prescription practice in patients of upper respiratory tract infection at a pediatric outpatient clinic in Punjab. Indian J. Pharm. Pract..

[35] Servia-Dopazo M., Taracido-Trunk M., Figueiras A. (2021). Non-clinical factors determining the prescription of antibiotics by veterinarians: A systematic review. Antibiotics.

[36] WHO Antimicrobial Stewardship Programmes in Health-Care Facilities in Low-and Middle-Income Countries: A WHO Practical Toolkit. https://www.who.int/publications/i/item/9789241515481.

[37] Bloch-Teitelbaum A., Lüde S., Rauber-Lüthy C., Kupferschmidt H., Russmann S., Kullak-Ublick G.A., Ceschi A. (2013). Medication wrong route administration: A poisons center-based study. Expert Opin Drug Saf..

[38] Tate A., Smallwood C. (2021). Comparing the efficiency of paper-based and electronic data capture during face-to-face interviews. PLoS ONE.

[39] Gates M.C., Holmstrom L.K., Biggers K.E., Beckham T.R. (2015). Integrating novel data streams to support biosurveillance in commercial livestock production systems in developed countries: Challenges and opportunities. Front. Public Health.

